# Uncoated vs. Antibiotic-Coated Tibia Nail in Open Diaphyseal Tibial Fracture (42 according to AO Classification): A Single Center Experience

**DOI:** 10.1155/2021/7421582

**Published:** 2021-10-14

**Authors:** Tommaso Greco, Luigi Cianni, Chiara Polichetti, Michele Inverso, Giulio Maccauro, Carlo Perisano

**Affiliations:** ^1^Department of Ageing, Neurosciences, Head-Neck and Orthopedics Sciences, Orthopedics and Traumatology, Fondazione Policlinico Universitario Agostino Gemelli IRCSS, Rome 00168, Italy; ^2^Orthopedics and Traumatology, Università Cattolica del Sacro Cuore, Rome 00168, Italy

## Abstract

Implant-associated infections remain one of the main problems in the treatment of open tibia fractures. The role of systemic antibiotic prophylaxis is now agreed and accepted; nevertheless, recent literature also seems to emphasize the importance of local antibiotic therapy at the fracture site. Several therapeutic strategies have been proposed to overcome this new need. Antibiotic-coated nails play crucial role in this, allowing both infection prevention and favoring the fracture stabilization. We describe the outcome of patients with open diaphyseal tibia fracture treated either with a standard uncoated nail or a gentamicin-coated nail from January 2016 to December 2018 at our second level emergency-urgency department. Primary outcomes were infection rate and bone union rate. Other outcomes reported are reoperation rate, time between injury and nailing, and safety of antibiotic nail. Numerical variables were tabulated using mean, standard deviation, minimum, maximum, and number of observations. Categorical variables were tabulated using number of observations. 23 patients treated with uncoated nail and 23 patients treated with antibiotic-coated tibia nail were included in the study and were evaluated for a minimum follow-up of 18 months. Among the 46 patients, 9 were Gustilo-Anderson type I, 21 type II, and 16 type III open fracture. Regarding the bone healing rate at 12 months, 16 fractures in the first group and 18 in the second were completely healed. 4 infections were found in the first group (3 superficial surgical site infection and 1 osteomyelitis) and 3 superficial infections in the second one. No adverse events have been recorded with antibiotic-coated nails. In this unicentric retrospective study observed no deep wound infections and good fracture healing in the use of antibiotic-coated nails. Antibiotic nails have been shown to play a role in the treatment of fractures in critically ill patients with severe soft tissue damage.

## 1. Introduction

Implant-associated infections remain one of the major problems in orthopedic and trauma surgery. They often require multiple aggressive treatments such as implant removal, surgical debridement, and long-term antibiotic therapy with long in-hospital stay resulting in higher social and healthcare costs [1].

Considering the hard consequences involving the patient's quality of life, it is strongly required to improve the prevention and treatment of the infections, mostly in traumatology. Elective orthopedic surgery usually has a low infection rate (0.7-4.2%) [2] that increases in patients with acute fractures, especially in those with open fractures. Among all long bone fractures, open tibia fractures showed the highest rate of infection (8.8%) compared to closed fracture (2%), due to more extensive comminution, segmental bone loss, poorer soft tissue coverage, and vascularization [3, 4].

The risk factors associated with a high infection rate are [5, 6] as follows:

- The severity of the soft tissues injury up to 14.4% in Gustilo-Anderson (GA) III grade (31% in GA IIIB and C types) [7, 8]
- The trauma mechanism
- Polytrauma and primary external fixation
- Obesity
- Diabetes mellitus
- Smoking
- Chronic disease

Intramedullary nailing is the gold standard for tibia shaft fracture stabilization (42 according AO classification) [9], due to the lower infection rate reported compared to both external fixation and plating [10, 11]. The systemic antibiotic administration is now widely accepted, since it reduces the absolute risk of early wound infections up to 60% [12]. Nevertheless, systemic antibiotic administration may have limited efficacy in decreasing the infection risk associated with the use of foreign metal implant. Indeed, bacteria can colonize the surface of the implant forming a biofilm (glycocalyx) that protects them from the systemic antibiotic action. In addition, systemically delivered antimicrobials may not reach adequate concentration at the desired site due to the vascular damage.

Hence the need to develop new strategies for prophylactic local administration of antibiotics. Several strategies for local antibiotic delivery have been developed, e.g., polymethylmethacrylate (PMMA) bone cement, PMMA beads, antibiotic-impregnated collagen sponges, polyglycolic acid, poly-DL-lactide (PDLLA) coated implants, or hydroxyapatite coatings [13–18].

The purpose of this study is to analyze the use of antibiotic-coated nails in a specific type of open tibia fracture (the 42 according to the AO classification) comparing the results obtained with the standard and the antibiotic-coated nails in terms of bone and soft tissue healing rate, infection rate, reoperation rate, difference in time between trauma and nailing (TTN), and hospital stay.

## 2. Materials and Methods

This study was a retrospective, nonrandomized study (level III). All patients with open tibia fracture who accessed the Emergency Room of our hospital from January 2016 to December 2018 were collected. Patients who met the following inclusion criteria were included in the study: open tibia fractures 42 according to the AO classification amendable for intramedullary nailing (evaluated on conventional radiographs in two planes including knee and ankle joints), a signed informed surgical treatment consent, and at least 18 months of clinical and radiological follow-up. Patients with open diaphyseal tibia fracture treated with plate and screws or external fixation were excluded.

The results were reviewed retrospectively using the patients' hospital and operation charts. Two authors (CP and TG) independently analyzed radiographs and clinical data.

After selecting the patients with the above inclusion criteria, we divided them into 2 groups: those treated with a standard tibia intramedullary nail (ETN—Expert Tibial Nail, DePuy Synthes) and those with a gentamicin-coated intramedullary tibia nail (ETN PROtect, DePuy Synthes).

The choice of the type of nail was led by the local conditions' criticality and therefore the risk of superinfection. Any choice was made by the operating surgeon assessing for each patient: the local and general conditions, the possible allergy and/or intolerance to gentamicin or other aminoglycosides, and the possible renal impairment.

All patients received standard pre- and postoperative antibiotic prophylaxis according to our institution's protocols.

Patient demographics including age, sex, kind of trauma, Injury Severity Score (ISS) [19], fracture type (Arbeitsgemeinschaft fur Osteosynthesefragen/Orthopaedic Trauma Association (AO/OTA) classification) [20], Gustilo-Anderson grade (GA) [21], time to nailing (TTN), eventual primary external fixation (EF), and implant characteristics were recorded. Open fractures were subdivided by the Gustilo-Anderson classification at the time of the initial debridement in the operating room.

The minimum follow-up period was 18 months to a maximum of 30 months. Follow-up visits and radiographs were performed at 1, 3, 6, and 12 months, barring complications. Conventional radiographs of the fractured limb in two planes (anteroposterior and lateral) with knee and ankle joints inclusion were performed for all patients. Evidence of bone union was determined by radiographic assessment of four cortices per patient. The fracture was considered completely healed when 3 or four cortices were consolidated and partially healed when 1 or 2 cortices were consolidated [22].

The indication to weight-bearing has taken into account both the clinical data (presence or absence of pain, soft tissue condition) and the radiographic data, encouraging early mobilization of the adjacent joints, and partial load with crutches was granted not until at least 3/4 weeks with a total weight-bearing at 8-10 weeks.

Data on adverse events to antibiotics (local or systemic) and infections were collected throughout the follow-up period. Infections were identified and classified by dividing them between surgical site infections (soft tissues) and deep infections (osteomyelitis) [23].

During the follow-up laboratory parameters were monitored, i.e., C-reactive protein, leukocyte count, and hemoglobin.

All procedures were performed following written informed patient consent and in accordance with the ethical standards of the institutional and national research committee and the 1964 Declaration of Helsinki. All patients have provided written consent for the processing of personal data and for the publication of this case series.

The study design was approved by the Orthopedic Department council and our school board and has been reviewed for epidemiological and statistical validation by the public health institute of our institution.

### 2.1. ETN PROtect

The ETN PROtect implant is a titanium alloy (titanium–6% aluminum–7% niobium) cannulated nail used for intramedullary fixation of tibia fractures. The fully resorbable antibiotic coating consists of an amorphous poly (D, L-lactide) (PDLLA) matrix containing gentamicin sulphate. Over 40% of the antibiotic is released within 1 h, 70% within 24 h, and 80% within 48 h after implantation. In the following weeks, the PDLLA coating is fully resorbed by hydrolytic degradation [6, 24].

The cost of an uncoated ETN nail in Italy is about 500 euros; instead, the price of the ETN PROtect is around 2500 euros.

### 2.2. Statistical Analysis

All patients were included in the analysis of infection, bone healing, and adverse event information. Numerical variables were tabulated using mean, standard deviation, minimum, maximum, and number of observations. Categorical variables were tabulated using number of observations.

## 3. Results and Discussion

The patients included in the study were 46, 23 treated with standard intramedullary tibia nailing (Expert Tibia Nail, Synthes) (see Table 1) and 23 underwent surgical treatment with ETN PROtect (see Table 2), with a minimum of 18-month follow-up. There were no significant demographic differences between the two groups (see Table 3). There was instead a significant difference in between the two groups about the severity of the fracture and the grade of the exposure (the highest GA grade was found in the antibiotic-coated nail group). A tendentially higher ISS was found in the nonantibiotic nail group compared with the antibiotic nails one (29.17 ± 16.17 SD vs. 24.04 ± 16.27 SD).

The mean age in the group of patients treated with ETN nail was 41.09 (DS 17.56), with 19 males and 4 females. Six patients had isolated tibial fractures, and 17 patients had polytrauma. According to the AO classification, 8 fractures were classified as type 42-A, 10 as type 42-B, and 5 as type 42-C. According to the Gustilo-Anderson classification system for open fractures, 7 fractures were grade I, 11 were grade II, and 5 were grade III (4 grade IIIA and 1 grade IIIB).

In the group of patients treated with the ETN PROtect nail, the average age was 45.81 (SD 19.13), with 18 males and 5 females. Polytraumas were 14 out of 23 patients. Based on the AO classification, 11 were 42-A type tibial shaft fractures, 9 type 42-B, and 3 type 42-C fractures. Classifying the exposition according to the Gustilo-Anderson system, the grade I fractures were 2, the grade II were 10, and 11 were grade III (of which 6 grade IIIA, 3 grade IIIB, and 2 grade IIIC).

In the standard nail (ETN) group, there were a total of 38 concomitant injuries or secondary diagnosis (abdominal/pelvic, thoracic, head and neck involvement, or other associated fractures in 14, 8, 9, and 7 patients, respectively), with a group average ISS (Injury Severity Score) of 29.17 ± 16.17 DS.

Instead, in the group of antibiotic-coated nails, the associated injuries were 34 (12 in the abdominal-pelvic area, 9 were other bone fractures, 7 in the head-neck area, and 6 in the thoracic area), with an average ISS of 24.04 ± 16.27.

**Figure 1:**
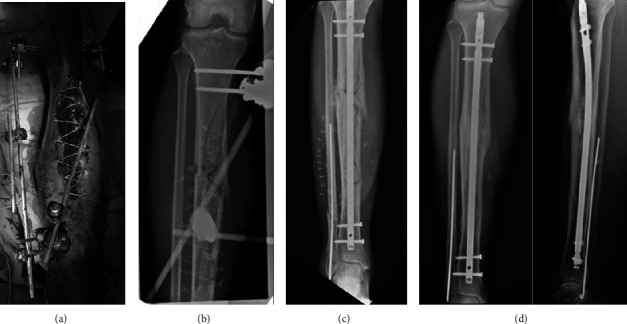
(a, b) A 47-year-old male polytrauma patient presented with an open, Gustilo grade IIIB, tibia fracture after a motor vehicle accident. Wound irrigation, debridement, and primary external fixation. (c) Nailing of tibia fracture with ETN PROtect. (d) Healing of the fracture 13 months after the injury.

All patients underwent sterile irrigation, wound debridement, and fracture stabilization within 6-8 h of arriving in the Emergency Room (Figure 1). Systemic prophylactic antibiotics were administered firstly on arrival in the Emergency Room, then right before surgery (a cephalosporin) and continued until the wound was closed according to the infectious disease specialist indications. In the uncoated nail group, 20 patients underwent temporary initial treatment with external fixation and subsequent definitive treatment with intramedullary nailing, while 3 patients underwent definitive nailing within 8 days of the trauma (1 to 5 days and 2 to 8 days). In the coated nail group (ETN PROtect), 19 patients were treated with temporary stabilization with external fixation; 4 patients underwent definitive intramedullary nailing within 5 days of the trauma instead (2 patients within 48 h and 2 patients at 5 days).

When necessary, plastic and reconstructive surgeon intervened from the early stages of the therapeutic process. Seven patients in the coated nail group and 4 of the uncoated group presented with severe soft tissue injuries and required soft tissue surgical treatment (e.g., vacuum therapy, skin grafting, and secondary skin closure).

**Figure 2:**
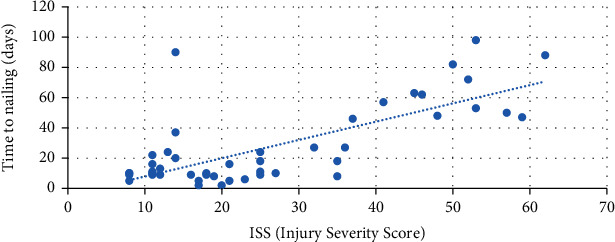
Correlation between ISS (Injury Severity Score) and time to nailing (TTN).

The average time between the temporary stabilization of the fracture and the definitive nailing (time to nailing (TTN)) was less in patients who received the antibiotic-coated nail compared to the standard nail (34.82 ± 37.86 vs. 21.55 ± 18.10 days; *P* = 0.7). This latter value appears to be quite long especially in the first group (34.82 days on average) being however strictly correlated to the patient's general complexity established through the ISS. A linear correlation links the ISS and the TTN (Figure 2).

### 3.1. Clinical and Radiographic Outcomes

**Figure 3:**
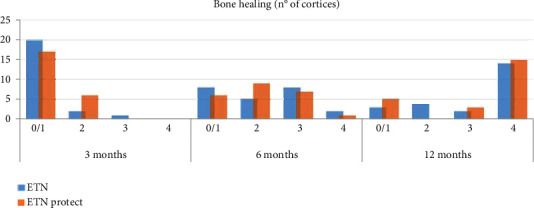
Analysis of bone healing at 3, 6, and 12 months in all patients of both groups (ETN vs. ETN PROtect) (0/1 cortices, 2 cortices, 3 cortices, 4 cortices).

Bone healing was assessed at radiographic follow-up considering the number of consolidated bone cortices on two plan conventional radiography. The data are reported in the chart bar (Figure 3).

At the 3-month follow-up, 20 patients for the ETN group and 17 for ETN PROtect group had 0-1 cortices consolidated. Patients with 2 healed cortices were 2 and 6 in the first and second groups, respectively. Only one patient in first group had 3 consolidated cortices, and none of the 46 patients showed consolidation of all 4 cortices.

At the 6-month radiographic follow-up, in the ETN group, there were 10 patients with complete healing (8 with 3 cortices and 2 with 4 cortices), while the remaining had 0/1 or 2 consolidated cortices. In the ETN coated group, there were 8 patients with 3 or 4 cortices consolidated.

At the 12 months after nailing radiographic follow-up, complete consolidation (3 or 4 cortices) of the fracture was observed in 16 patients treated with ETN and in 18 treated with ETN PROtect.

Six patients in the group of uncoated ETN showed a delayed consolidation and one an infected nonunion. In the group of ETN PROtect, 3 patients had a delayed consolidation and 2 nonunion.

Therefore, 6 patients in the first group and 3 in the second group required dynamization for delayed healing.

At the last study follow-up, complete bone healing was achieved in 22 patients in the first group and in all 23 in the second group.

**Figure 4:**
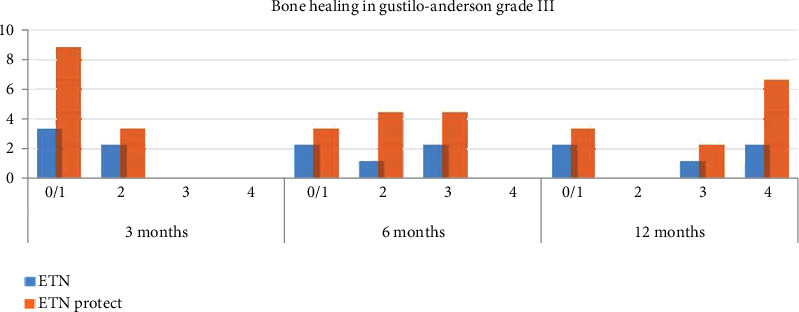
Analysis of bone healing at 3, 6, and 12 months in subgroup of Gustilo-Anderson type III open fracture (ETN vs. ETN PROtect) (0/1 cortices, 2 cortices, 3 cortices, 4 cortices).

Analyzing the healing process considering only the subgroup of patients with GA grade III fractures (Figure 4), it emerges that at the 3-month follow-up among the 5 patients treated with standard ETN 3 had 0/1 consolidated cortices, and the other 2 patients showed 2 consolidated cortices. In the ETN coated group instead, 8 out of the 11 patients had 0/1 healed cortices, and 3 patients had 2 healed cortices. At the 6-month follow-up, the number of healed cortices was 0/1 in 2 patients, 2 in 1 patient, and 3 in 2 patients for the standard nail group; in the group of antibiotic-coated nails, 3 patients had 0/1 consolidated cortices, 4 patients 2 cortices, and 4 other patients 3 cortices.

At the 12-month follow-up, bone consolidation (3/4 cortices) was achieved by 3 of 5 patients in the standard nail group and 8 of 11 in the antibiotic ETN group.

There are no statistically significant differences concerning the bone healing rate in the 2 groups. The only statistically significant difference is in the average number of consolidated bone cortices at the 6-month follow-up in the subgroup of the GA grade III fractures, where more advanced healing seems to be appreciated in the antibiotic-coated nail group (1.44 ± 1.13 DS vs. 2.80 ± 0.84 DS; *P* = 0.03) (see Table 4).

Considering the 46 patients included in the study, 7 infections were found (of whom 4 in the group of patients treated with uncoated nail and 3 in the ETN PROtect group). As regards the 4 cases of the first group, 3 were superficial surgical site infections (1 GA grade II fracture, 1 GA IIIA fracture, and 1 GA IIIB fracture) treated with surgical wound debridement and targeted antibiotics based on intraoperative microbiological swab results. The fourth patient (with Gustilo-Anderson IIIA fracture), with osteomyelitis and infected nonunion, underwent Masquelet procedures [25].

All 3 patients in the second group with superficial surgical site infection (respectively, GA grades II, IIIA, and IIIC) required a second intervention for wound debridement and subsequent targeted antibiotic therapy.

In the first group, we observed a total of 10 reoperation (6 dynamizations of nail, 3 wound debridement, and 1 revision of infected nonunion according to Masquelet technique) and 6 in the second group of antibiotic-coated nails (3 dynamizations and 3 superficial wound debridement) (see Table 5).

Throughout our follow-up, we did not observe local or systemic adverse effects to gentamicin. Furthermore, no antibiotic-coated nail-related side effects were observed either on the bone or on the soft tissue healing process.

## 4. Discussion

Despite the progress achieved in the management of traumatized patients, wound infection and osteomyelitis remain a major issue in the fracture treatment, especially in open fractures of the tibia.

The latest systematic review and meta-analysis by Craig et al. [7] has shown the benefit of local prophylactic antibiotic therapy, in addition to systemic antibiotics, due to reduce the infection rate in open tibia fractures treated with intramedullary nailing.

In this perspective, we analyzed our first results concerning the group of patients with open tibia fracture treated with the antibiotic-coated nail (gentamicin). Among the first studies in literature on this matter, Fuchs et al. [26] did not observe infections and reported good clinical results in his case series of 21 patients. Metsemakers et al. [6] did not report infections in his series of 16 patients (11 acute fractures and 5 revision cases). However, current literature is lacking in studies comparing the two different types of intramedullary nail in the treatment of open tibia fractures. Pinto et al. reported the protective role of antibiotic-coated nails in the treatment of open tibial fractures compared to standard nails first, by analyzing however only Gustilo I and II fractures [27]. Our paper presents two large and varied groups of complex patients in terms of general conditions (high percentage of polytrauma) and with a high percentage of open type III fractures according to Gustilo-Anderson.

We reported 4 cases of infection in patients treated with nonantibiotic nails (including 3 superficial surgical site infections and one infected nonunion) and 3 infections in patients treated with antibiotic-treated nails (all superficial surgical site infections). Furthermore, the careful data analysis of the patients with grade III open fracture according to the Gustilo-Anderson classification showed that infections have a higher trend in the group of nonantibiotic nails (3 patients out of 5) compared to the group of antibiotic nails (2 out of 11), although without statistical significance given the size of the sample. In addition, the infections found in the group of patients treated with antibiotic nails were all superficial infections, while in the group of nonantibiotic nails there was instead also a case of bone infection (osteomyelitis). Considering once more the 4 patients that underwent infection in the nonantibiotic nail group, with reference to the demographic characteristics and the fracture type (2 patients GA IIIA and one GA IIIB), it seems that the infection risk is driven more by local conditions than by the general criticality of the patient.

As far as bone healing is concerned, there does not seem to be any significant difference in the two groups, underlining that the local antibiotic pharmacodynamics does not interact with the bone and soft tissue healing. On the other hand, analyzing the subgroup of GA III fractures, there appears to be a higher healing rate at 6 months with statistical significance.

The absence of both local and systemic side effects with antibiotic nails is in line with the available literature [28]. It is difficult to compare our data with the available literature, given the heterogeneity of the systems used to classify the infections in the various studies and the severity of the traumas considered (in our study, the percentage of patients with high grade fracture according to Gustilo-Anderson is high especially in the group of antibiotic nails).

### 4.1. Limits of Our Study

The strengths of our study are undoubtedly the long follow-up, the homogeneity of the sample (all 42 fractures classified according to AO classification), and the high percentage of patients with severe grade of open fractures (according Gustilo-Anderson classification). Limits are the retrospective analysis, the lack of randomization, and the size of the sample to obtain a statistical significance. The small cohort of patients does not allow for adequate statistical analysis and definitive evaluations on the incidence of infection, on the rate of reoperation, and on adverse events but, at the present time, it represents the largest case history available in the literature. Another consideration to make concerns the use of a tibia nail coated with gentamicin; the choice fell on this type of implant as it is the only one currently available on the market.

Despite these limitations, our study suggests a role of antibiotic-coated nails for the prevention of deep (implant-related) infections especially in the most serious fractures, although future large-scale randomized clinical trials are needed to achieve results with statistical significance.

## 5. Conclusions

This study shows that gentamicin-coated intramedullary nails might play a role in the treatment of high-grade open tibia fractures. Although the number of patients and infections is too low to draw firm conclusions, the lack of severe infections or other adverse events in this retrospective study is a promising starting point to further investigate the protective effect of antibiotic-coated implants against fracture-related infections. This study urges the need for sufficiently powered comparative studies, preferably in a prospective and randomized fashion. Especially since no real alternatives exist.

## Figures and Tables

**Table 1:** Patient and implant characteristics of ETN group (ISS: Injury Severity Score; EF: external fixation; ^#^bone consolidation in more of 12 months).

N°	Age	Sex	Fracture type (AO)	Gustilo type	Polytrauma	ISS	EF	Nail length ^#^ diameter (mm)	Infection	Bone union
1	37	M	42B3	3A	Yes	41	Yes	345 ^#^ 10	No	Yes
2	82	M	42A2	3B	Yes	52	Yes	330 ^#^ 11	Yes	Yes
3	23	M	42C2	2	Yes	32	Yes	330 ^#^ 9	No	Yes^#^
4	57	M	42B2	2	Yes	53	Yes	330 ^#^ 9	No	Yes
5	17	M	42B2	2	Yes	62	Yes	330 ^#^ 12	No	Yes
6	21	M	42B2	3A	Yes	46	Yes	345 ^#^ 10	Yes	No
7	20	M	42B2	1	No	12	Yes	360 ^#^ 9	No	Yes^#^
8	32	F	42A3	3A	Yes	50	Yes	345 ^#^ 8	Yes	Yes
9	55	F	42C3	2	Yes	35	Yes	300 ^#^ 10	Yes	Yes^#^
10	51	M	42A2	1	No	19	No	300 ^#^ 10	No	Yes
11	46	F	42C3	2	Yes	18	Yes	300 ^#^ 9	No	Yes
12	19	M	42B2	1	No	13	Yes	380 ^#^ 10	No	Yes^#^
13	54	M	42B2	1	Yes	21	Yes	360 ^#^ 9	No	Yes^#^
14	51	M	42C3	2	Yes	45	Yes	345 ^#^ 10	No	Yes
15	29	F	42A3	2	Yes	8	No	280 ^#^ 9	No	Yes^#^
16	22	M	42A3	1	Yes	16	Yes	375 ^#^ 10	No	Yes
17	38	M	42A3	2	No	11	Yes	330 ^#^ 9	No	Yes
18	54	M	42B2	2	No	11	Yes	315 ^#^ 9	No	Yes
19	45	M	42C2	1	Yes	27	Yes	345 ^#^ 10	No	Yes
20	64	M	42A3	1	Yes	25	Yes	340 ^#^ 10	No	Yes
21	64	M	42A3	3A	Yes	35	Yes	340 ^#^ 10	No	Yes
22	29	M	42B2	2	No	14	Yes	340 ^#^ 10	No	Yes
23	35	M	42B2	2	Yes	25	No	300 ^#^ 11	No	Yes

**Table 2:** Patient and implant characteristics of ETN PROtect group (ISS: Injury Severity Score; EF: external fixation; ^#^bone consolidation in more of 12 months).

N°	Age	Sex	Fracture type (AO)	Gustilo type	Polytrauma	ISS	EF	Nail length ^#^ diameter (mm)	Infection	Bone union
1	20	M	42B3	2	Yes	36	Yes	360 ^#^ 11	No	Yes
2	78	M	42A1	2	No	11	Yes	315 ^#^ 11	No	Yes
3	58	M	42A3	2	Yes	25	Yes	375 ^#^ 13	No	Yes^#^
4	18	M	42B3	2	Yes	21	No	375 ^#^ 10	No	Yes
5	68	F	42B2	3A	Yes	57	Yes	315 ^#^ 11	No	Yes
6	22	M	42A3	1	Yes	17	No	360 ^#^ 10	No	Yes
7	75	M	42A1	3B	No	8	Yes	315 ^#^ 10	No	Yes
8	63	M	42A2	3A	No	14	Yes	330 ^#^ 11	No	Yes^#^
9	29	M	42B3	3A	Yes	53	Yes	345 ^#^ 11	No	Yes
10	30	M	42C3	2	Yes	23	Yes	340 ^#^ 9	No	Yes^#^
11	28	M	42B3	2	No	8	Yes	345 ^#^ 8	No	Yes
12	52	F	42B3	2	No	11	Yes	330 ^#^ 11	No	Yes
13	43	M	42A2	2	No	14	Yes	375 ^#^12	No	Yes
14	30	M	42B2	2	No	8	No	315 ^#^ 10	No	Yes
15	57	M	42A2	3A	No	12	Yes	360 ^#^ 10	Yes	Yes
16	22	M	42A3	3C	No	11	Yes	345 ^#^ 10	Yes	Yes^#^
17	62	M	42C3	2	Yes	18	Yes	330 ^#^ 9	Yes	Yes
18	50	M	42A1	3C	Yes	48	Yes	340 ^#^ 11	No	Yes
19	45	F	42A2	3B	Yes	59	Yes	285 ^#^ 9	No	Yes^#^
20	65	F	42A2	1	Yes	20	No	315 ^#^ 10	No	Yes
21	47	M	42C3	3A	Yes	37	Yes	375 ^#^ 10	No	Yes
22	45	F	42B3	3B	Yes	17	Yes	315 ^#^ 10	No	Yes
23	50	M	42B2	3A	Yes	25	Yes	330 ^#^ 11	No	Yes

**Table 3:** Comparison of patient characteristics and outcome in the 2 groups.

	ETN (23)	ETN PROtect (23)
Age (mean, DS)	41.09 ± 17.56	45.81 ± 19.13
Gender		
Male	19	18
Female	4	5
Type of fracture		
42A	8	11
42B	10	9
42C	5	3
Gustilo type		
I	7	2
II	11	10
III	5 (4 IIIA; 1 IIIB)	11 (6 IIIA; 3 IIIB; 2 IIIC)
Polytrauma		
Yes	17	14
No	6	9
ISS (mean, DS)	29.17 ± 16.17	24.04 ± 16.27
Concomitant injuries		
Other fractures (femur; humerus; pelvic ring; cervical/thoracic/lumbar/sacral vertebrae; clavicle; foot/hand bones fracture)	7	9
Head/neck (mandibular or maxillary fractures; facial bone fracture; brain contusion; subarachnoid hemorrhage; epidural hematoma)	9	7
Chest (rib contusion; rib/sternum fractures; lung/pericardium contusion; hemopericardium, hemothorax, pneumothorax)	8	6
Abdomen and pelvic contents (hepatic, splenic, or renal contusion; spleen rupture/laceration; retroperitoneal hematoma/hemorrhage; peritoneal laceration; intra-abdominal/intrapelvic major bleeding; scrotum rupture; urethral laceration)	14	12
Days injury to nailing (TTN) (mean, DS)	34.82 ± 37.86	21.55 ± 18.10
Plastic surgery (skin grafting and secondary skin closure)	4	7
Infection	4	3
Superficial infection	3 (GA grade II, IIIA, IIIB)	3 (GA grade II, IIIA e IIIC)
Osteomyelitis	1 (GA grade IIIA)	—
Bone union at 12 months		
Healed	16	18
Partially healed	6	5
Infected nonunion	1	—
Bone union after 12 months (after dynamization)	22	23

**Table 4:** Bone healing at 3, 6, and 12 months. Analysis of bone healing in the subgroup of GA III fracture.

	3 months (*n* of cortices, DS)	6 months (*n* of cortices, DS)	12 months (*n* of cortices, DS)
ETN (23)	0.71 ± 0.78	1.86 ± 1.11	3.24 ± 1.51
ETN PROtect (23)	0.83 ± 0.78	2.26 ± 1.05	3.39 ± 1.16
*P*	0.6	0.2	0.7
Gustilo-Anderson III			
ETN (5)	0.78 ± 0.97	1.44 ± 1.13	2.78 ± 1.86
ETN PROtect (11)	1.40 ± 1.14	2.80 ± 0.84	3.00 ± 0.84
*P*	0.3	0.03	0.1

**Table 5:** Number and type of reoperation.

	ETN group	ETN PROtect group
Dynamization for delayed healing	6	3
Wound debridement	3	3
Infected nonunion	1	—

## Data Availability

The study data will be available upon request to the corresponding author (email: tommaso.greco01@icatt.it).
